# Topical use of tranexamic acid can effectively decrease hidden blood loss during posterior lumbar spinal fusion surgery: A retrospective study

**DOI:** 10.1097/MD.0000000000008233

**Published:** 2017-10-20

**Authors:** Zhinan Ren, Shugang Li, Lin Sheng, Qianyu Zhuang, Zheng Li, Derong Xu, Xin Chen, Pengxiang Jiang, Xiao Zhang

**Affiliations:** Department of Orthopaedics, Peking Union Medical College Hospital, Chinese Academy of Medical Sciences and Peking Union Medical College, Beijing, China.

**Keywords:** hematocrit, hidden blood loss, lumbar spinal fusion, tranexamic acid, wound drainage

## Abstract

In spinal fusion surgery, total blood loss (TBL) is composed of visible blood loss from the surgical field and wound drainage, and hidden blood loss (HBL). Until now, no published studies exist reporting the effect of topical use of tranexamic acid (tTXA) on HBL in patients undergoing posterior lumbar spinal fusion surgery. This study aimed to explore the effect of tTXA on HBL during primary posterior lumbar spinal fusion surgery. Between September 2014 and September 2016, 100 adult patients (age > 18 years) with lumbar disc herniation or lumbar spinal stenosis undergoing primary posterior lumbar instrumented spinal fusions at 1 institution were divided into tTXA and control groups. The primary outcome was HBL. Secondary outcomes include TBL, intraoperative blood loss (IBL), postoperative blood loss (PBL), hemoglobin (HGB) levels on preoperative (Pre-op) and postoperatively (days 1–3, POD1, POD2, POD3, respectively), and amount of allogeneic blood transfusion. Complications occurring perioperatively until hospitalization discharge were also collected. In the tTXA group (n = 50 patients), wound surface was soaked with TXA (1 g in 100 mL saline solution) for 5 minutes before wound closure. For the control group (n = 50 patients), wound surface was soaked with the same volume of normal saline. There were no significant differences in demographics, surgical traits between the 2 groups. There were no significant differences in IBL or perioperative blood transfusion requirements between the 2 groups. However, in the tTXA group, TBL, PBL, and HBL were significantly lower than those in the control group (550 ± 268 vs 833 ± 298 mL, 53.5 ± 43.9 vs 136.7 ± 87.9 mL, 356.7 ± 254.8 vs 501.1 ± 216.9 mL, *P* < .001, respectively). HGB levels were significantly higher in the tTXA group (*P* < .001) on POD1 and had a slower decline on POD2 and POD3 than the control group. No complications associated with TXA were observed. From these data, we conclude that tTXA can effectively reduce HBL, without significant complications in adult patients undergoing posterior lumbar spinal fusion surgery.

## 1 Introduction

Spinal surgery may be associated with significant blood loss, especially in multilevel procedures and remains a challenge.^[1]^ When assessing total blood loss (TBL) during spinal fusion surgery, the standard is to measure intraoperative blood loss (IBL) and postoperative blood loss (PBL), which ignores so-called “hidden blood loss” that has been found to be significant in the field of surgery.^[2,3]^ Hidden blood loss (HBL) negatively affects patients’ outcomes, such as medical complications and prolonged hospitalization time. The percentage of HBL was reported to be approximately 45% of TBL, according to the reports of Smorgick et al.^[2]^ HBL is a significant portion of TBL in the patients after spinal fusion surgery, which can result in the increased allogenic blood transfusion requirements.^[4,5]^ Potential problems associated with allogeneic blood transfusion include disease transmission, transfusion reactions, and infections.^[6]^ Therefore, how to reduce blood loss and transfusions of spine surgery has become an urgent problem to be solved.

As a kind of antifibrinolytic agent, tranexamic acid (TXA) is a synthetic lysine analogue that interferes with fibrinolysis through binding reversibly and competitively to lysine-binding domains on plasminogen, plasmin, and tissue plasminogen activator.^[7]^ Comparing with intravenous use of TXA (ivTXA), tTXA has the advantages of easy to administer, providing a maximum concentration of TXA at the bleeding site with little or no systemic exposure of TXA. Therefore, tTXA can potentially avoid the complications of ivTXA such as venous thrombosis, renal impairment, and convulsive seizures. Although the concept of HBL was put forward since 2000, scarce published studies have examined the effect of tTXA on HBL in patients undergoing spinal fusion surgery; we performed a retrospective study to determine whether tTXA can reduce HBL in patients undergoing posterior lumbar spinal fusion procedures. In addition, we observed the perioperative complications that may be associated with the use of TXA.

## 2 Methods

### 2.1 Patient profile

A retrospectively nonrandomized case–control study was conducted. We analyzed the clinical data of patients with lumbar disc herniation (LDH) or lumbar spinal stenosis (LSS) who underwent primary posterior lumbar instrumented spinal fusions from September 2014 to September 2016. Fifty consecutive patients treated with tTXA were collected first. Then, we screened another 50 patients who just did not receive treatment of TXA to match baseline data. The study was approved by the Ethics Committee of Peking Union Medical College Hospital. The Ethics Committee specifically approved that not informed consent was required because data were going to be analyzed anonymously and due to the retrospective nature of the study. One hundred adult patients (age >18 years) were enrolled in the study. Exclusion criteria include preoperative anemia (i.e., hemoglobin < 110 g/L in females; hemoglobin < 120 g/L in males), preoperative anticoagulant or antiplatelet therapy within three months, coagulopathy, preoperative platelet count (PLT) < 100 × 10^9^/L, international normalized ratio (INR) >1.4, prolonged activated partial thromboplastin time (APTT) >1.4 × normal, a history of thromboembolic disease, and female patients in the menstrual period. If intraoperative surgical complications (such as uncontrollable surgical bleeding or dural tears, etc.) occurred, the patients would be excluded from the study to provide a more homogenous group. Patients with very large blood losses with more than 1500 mL were also excluded to minimize the effect of hemodilution on the calculation.^[8]^

### 2.2 Surgical procedures

All patients were positioned prone on the operating table with the abdomen free. The anesthetic technique in all patients was similar. Operative procedures were performed by the same spine surgeon. All patients underwent fusion with pedicle screws and rod instrumentation. Laminectomy, partial resection of the facet, and a foraminotomy were performed on all patients. In all cases, posterolateral bone graft fusion was done. The bone graft for fusion was a mixture of allogeneic bone and autologous bone obtained from the decompression procedure. Then, the incision was rinsed, and hemostasis by radiofrequency bipolar was accomplished. After that, in the tTXA group, wound surface was soaked with TXA (1 g in 100 mL saline solution) for 5 minutes before wound closure. In the control group, wound field was soaked with the same volume of normal saline. A negative-pressure drainage was placed, and a layer-to-layer suture was carried out to close the wound. Cellsaver equipment was not used in this study.

### 2.3 The guidelines for transfusion

Packed red blood cells (RBCs) were given 1 U at a time if the hemoglobin concentration < 70 g/L, or at a higher hemoglobin concentration if continuing blood loss was occurring. The criteria for transfusing fresh frozen plasma (FFP, 200 mL at a time) was INR >1.5, or APTT >1.5 × normal baseline with continuing bleeding. The criteria for transfusing platelets (1 U at a time) were PLT < 100 × 10^9^/L, with continuing bleeding.^[9]^ If the surgeon considered it clinically unsafe to withhold blood transfusion, blood products were given.

### 2.4 Evaluation

The primary outcome was HBL. Secondary outcomes include TBL, IBL, PBL, HGB levels pre- and postoperatively, and amount of allogeneic blood transfusion, including RBCs, FFP, and platelets administered during the hospitalization. The blood loss was determined on the basis of milliliters. The amount and hematocrit (Hct) levels of drainage were measured for 24 and 48 h, respectively. Twenty-four hour drainage < 50 mLwas defined as the standard for removal of drainage tube. HGB were tested preoperatively (Pre-op), and at multiple times postoperatively (days 1–3, POD1, POD2, POD3, respectively). Complications occurring perioperatively through hospitalization discharge were also collected. Every patient was given venous Doppler ultrasonography examination routinely by senior ultrasound doctors for screening before they were discharged. The incidence of thromboembolic events was tracked for 3 months after surgery.

### 2.5 Calculation

As the blood loss is continuing, the patient's circulating volume is maintained by the fluid administered perioperatively and the simultaneous shift of fluid into the circulating compartment. The blood volume was assumed to be constant before and after surgery.

TBL was calculated by the Gross formula,^[10]^ which is

TBL = patient's blood volume (PBV) × (Hct_pre_ - Hct_post_)/Hct_ave._

Hct_pre_ = the preoperative Hct level; Hct_post_ = the Hct on the morning of POD3, by this time, the patients were hemodynamically stable and thus fluid shifts would have been largely completed. Hct_ave_ = the average of the Hct_pre_ and Hct_post._

The PBV (L) was assessed according to the formula of Nadler et al^[11]^:

PBV = k1 × height (m)^3^ + k2 × weight (kg) + k3 (k1 = 0.3669, k2 = 0.03219, and k3 = 0.6041 for men;k1 = 0.3561, k2 = 0.03308, and k3 = 0.1833 for women).

PBL was measured from wound drainage, which was converted to whole blood volume for each patient, using their Hct of drainage and Hct_ave_, which is

PBL = (WD_POD1_ × Hct_POD1_+WD_POD2_ × Hct_POD2_)/Hct_ave._(WD_POD1_ = the volume of wound drainage on POD1; Hct_POD1_ = the Hct of wound drainage on POD1; WD_POD2_ = the volume of wound drainage on POD2; Hct_POD2_ = the Hct of wound drainage on POD2;Hct_ave_ = the average of the Hct_pre_ and Hct_post_).

If allogeneic transfusion was given, TBL is equal to the loss calculated from the change in Hct and the volume transfused. IBL was estimated by weighing surgical sponges, measuring blood collected by suction canisters, and subtracting all irrigations fluids added to the surgical field. As the primary outcome, HBL = TBL - IBL - PBL. Although the HGB was not used in the calculations, the fall in HGB illustrates the blood loss that commonly occurs since the postoperative period.

### 2.6 Data analysis

All statistical analyses were performed with SPSS statistical software (version 23.0; SPSS, Chicago, IL). Data were represented in mean ± standard deviation (mean ± SD) and frequency (percentage). Statistical differences between the groups were compared using the Chi-square test for categorical variables and Student *t* test (equal variance groups with parametric data) for continuous variables appropriately. Two-sided value of *P* < .05 was considered statistically significant.

## 3 Results

### 3.1 Baseline characteristics

One hundred patients, 61 females and 39 males, aged 28 to 82 years, were included in the study. There are 50 patients in the tTXA group and 50 patients in the control group. All patients had primary posterior lumbar spinal decompression and instrumented fusions. Mean of patients’ age was 55.2 ± 13.0 years and BMI was 25.7 ± 2.8 in the tTXA group and 58.7 ± 12.9 years and 25.1 ± 3.1 in the control group, respectively. There were no significant differences in the demographics (gender, age, BMI) and surgical characteristics (surgical segment, length of incision, and duration of operation) between the 2 groups (Table 1).

**Table 1:**
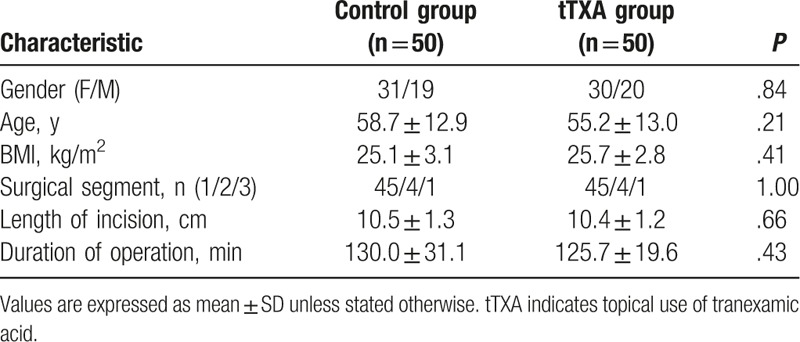
Demographic values.

### 3.2 Blood loss

**Figure 1:**
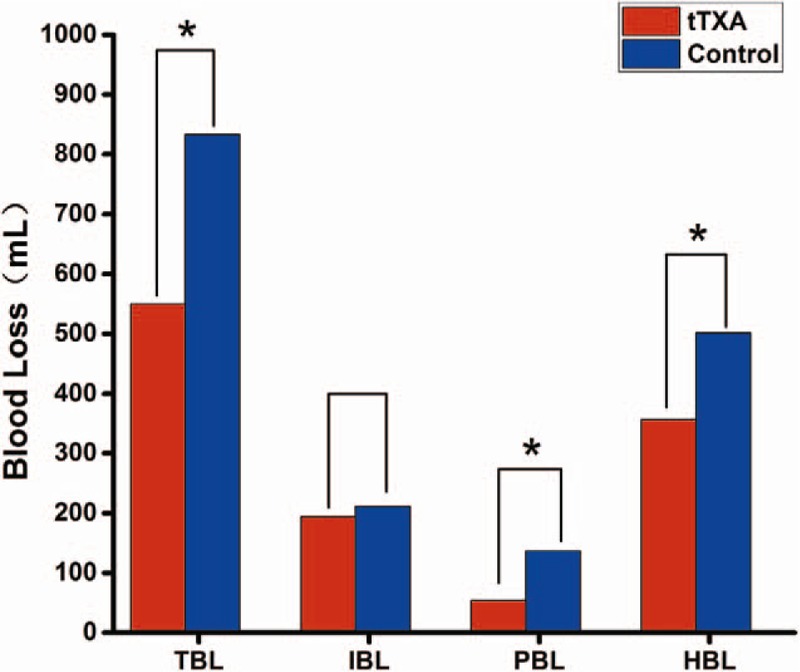
TBL indicates total blood loss. IBL indicates intraoperative blood loss. PBL indicates postoperative blood loss. HBL indicates hidden blood loss. The asterisk indicates values that were significantly different between the groups.

The outcomes of blood loss are summarized in Fig. 1. There was no significant difference in IBL. However, in the tTXA group, the TBL, PBL, and HBL were significantly lower than those in the control group (550 ± 268 vs 833 ± 298 mL, 53.5 ± 43.9 vs 136.7 ± 87.9 mL, 356.7 ± 254.8 vs 501.1 ± 216.9 mL, *P* < .001, respectively). No patient received allogeneic blood transfusion perioperatively in the 2 groups.

### 3.3 HGB levels on pre-op and POD 1, 2, 3

**Figure 2:**
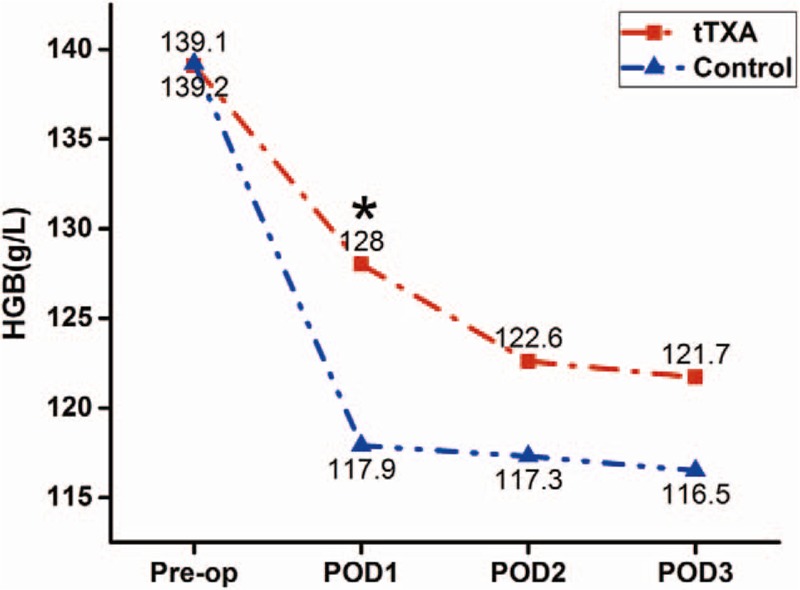
The pre- and postoperative HGB. The asterisk indicates values that were significantly different between the groups. HGB = hemoglobin.

The changes of HGB levels in perioperative period are shown in Fig. 2. Preoperative HGB was similar between the 2 groups. However, HGB levels were significantly higher in the tTXA group (128 ± 16 vs 117 ± 16 g/L, *P* = .006) on POD1. No significant differences on POD2 and POD3, but tTXA-treated patients can gain a slower decline of HGB than the control group.

### 3.4 Complications

No postoperative cases of myocardial infarction (MI), cerebral vascular accident (CVA), deep vein thrombosis (DVT), pulmonary embolism (PE), wound complications, or hematoma formation within the spinal canal were found in all patients. None of the patients developed side effects related to TXA (i.e., seizures, nausea, diarrhea, renal failure).

## 4 Discussion

The main finding in this comparative study revealed that the tTXA group had a remarkable decreased volume of PBL (53.5 ± 43.9 mL with tTXA vs 136.7 ± 87.9 mL without, *P* < .001), which was in agreement with the results from Krohn et al,^[12]^ suggesting that tTXA can remarkably reduce PBL in spinal fusion surgery. However, none of the aforementioned authors have taken HBL into account and measured the effect of tTXA on HBL. In the tTXA group of our study, HBL and TBL were significantly lower than that in the control group (356.7 ± 254.8 vs 501.1 ± 216.9 mL, 550 ± 268 vs 833 ± 298 mL, *P* < .001, respectively), suggesting that tTXA can remarkably decrease HBL in spinal fusion surgery. It chiefly works by attenuating the binding capacity of plasminogen and tissue plasminogen activator to fibrin, thereby decreasing the subsequent conversion of plasminogen to the enzymatically active serine protease plasmin, which leads to dissolution of fibrin clots.^[13]^ Furthermore, HGB levels were significantly higher in the tTXA group (128 ± 16 vs 117 ± 16 g/L, *P* = .006) on POD1. In spite of no significant differences being found on POD2 and POD3, tTXA-treated patients can gain a slower decline of HGB than the control group. Unfortunately, postoperative blood transfusion did not differ between the tTXA and control groups. Given that the surgical segments are mostly single-level, which have relatively small volume of blood loss in the patients of this study, it is underpowered to examine the assumption that tTXA can decrease the amount of blood transfusion.

Our study demonstrates that posterior fusion of the spine involves a substantial HBL, which is not recognized by the usual practice of assessing the IBL and PBL. This study showed that the HBL in control group was 501.1 ± 216.9 mL, nearly 58% of the TBL, whereas the volume of HBL in tTXA group was 356.7 ± 254.8 mL, approximately 55% of the TBL, and both were more than the results reported by Smorgick et al,^[2]^ which were 45%. The variations in the Hct of wound drainage may be the sources of error. In most of current studies, PBL was calculated by measuring the amount of “blood” by wound drainage. However, the Hct levels of drainage were significantly lower than the serum level, indicating that the postoperative drainage included not only blood but also tissue fluid exudation.^[14]^ Consequently, the volume of wound drainage is not equivalent to the PBL. Therefore, the previous authors simply measure the volume of PBL by wound drainage may overestimate the PBL, thereby underestimating the HBL. Given this, in our study, PBL was measured from wound drainage, which was converted to whole blood volume for each patient, using their hematocrit of drainage and Hct_ave_. We thought that our results were more accurate than the previous.

The pathological mechanism of HBL remains to be elucidated. Previous studies provided some possible explanations for the mechanisms of HBL, such as hemolysis^[15,16]^ and loss going into tissue compartments.^[17]^ Besides, it was proposed that the HBL may be, at least in part, due to the residual blood entering the dead space in spinal fusion surgery. Spine vertebras are rich in blood supply. When the decompression procedures were performed, bone cuts may result in significant bleeding possibly continuing for many hours even after completion of the operation. In addition, the internal fixation systems consisting of pedicle screws and rod instrumentation also provide storage cavities for HBL. However, the mechanisms and influential factors for HBL following spinal surgery are not fully clear, and some of them are still controversial. However, it is clear that we should put great emphasis on the estimation of HBL as an important part of total perioperative blood loss. Safety of tTXA was very important and should be focused on. There were no cases of MI, CVA, DVT, PE, or hematoma formation within the spinal canal between the 2 groups in our study. No patient experienced complications from the TXA, including seizures, wound complications, nausea, diarrhea, and renal failure.

The strength of our study is the homogeneity of the groups in a population at risk for significant blood loss. This was a series of adult patients having primary posterior lumbar spinal fusions by the same surgeon at the same hospital. Last but not the least, the primary outcome in our study was HBL, which was not explored well in other literatures. In addition, the transfusion criteria we used were strict. One of the limitations of this study is that it is not powered to examine the assumption that tTXA can decrease the requirements of blood transfusion. Therefore, rigorous prospective randomized controlled trials should be undertaken in groups of patients undergoing multilevel instrumented procedures or more complex spinal surgery such as pedicle subtraction osteotomy or vertebral column resection surgery to further assess the efficacy and safety of tTXA for spinal fusion surgery.

## 5 Conclusion

This study demonstrates that there is a substantial HBL in adult patients having primary posterior lumbar spinal fusion surgery. tTXA can effectively reduce HBL without increasing the risk of DVT, PE, and other complications.
